# Supplementary material for: Short-term exposure to intermittent hypoxia leads to changes in gene expression seen in chronic pulmonary disease
Source: eLife. 2021 Feb 18;10:e63003. doi: 10.7554/eLife.63003 (PMC7909952; doi:10.7554/eLife.63003)
Supplement: Transparent reporting form [file elife-63003-transrepform.docx]

***eLife’s* transparent reporting form**

We encourage authors to provide detailed information *within their submission* to facilitate the interpretation and replication of experiments. Authors can upload supporting documentation to indicate the use of appropriate reporting guidelines for health-related research (see [EQUATOR Network](http://www.equator-network.org/%20)), life science research (see the [BioSharing Information Resource](https://biosharing.org/)), or the [ARRIVE guidelines](http://www.plosbiology.org/article/info:doi/10.1371/journal.pbio.1000412) for reporting work involving animal research. Where applicable, authors should refer to any relevant reporting standards documents in this form.

If you have any questions, please consult our Journal Policies and/or contact us: [editorial@elifesciences.org](mailto:editorial@elifesciences.org).

**Sample-size estimation**

- You should state whether an appropriate sample size was computed when the study was being designed
- You should state the statistical method of sample size computation and any required assumptions
- If no explicit power analysis was used, you should describe how you decided what sample (replicate) size (number) to use

Please outline where this information can be found within the submission (e.g., sections or figure legends), or explain why this information doesn’t apply to your submission:

We did not use sample size computation nor power analysis. Considering the importance of biological replicates, we decided to use as many replicates as we can. For the bulk RNA-seq, six biological replicates per group were applied. For the single cell RNA-seq, we have 3 independent biological replicates per group and sufficient number of cells (~28K) in total. This information can be found in the Materials and Methods, figure legends and supplementary files.

**Replicates**

- You should report how often each experiment was performed
- You should include a definition of biological versus technical replication
- The data obtained should be provided and sufficient information should be provided to indicate the number of independent biological and/or technical replicates
- If you encountered any outliers, you should describe how these were handled
- Criteria for exclusion/inclusion of data should be clearly stated
- High-throughput sequence data should be uploaded before submission, with a private link for reviewers provided (these are available from both GEO and ArrayExpress)

Please outline where this information can be found within the submission (e.g., sections or figure legends), or explain why this information doesn’t apply to your submission:

• Bulk RNA-seq experiments included 6 biological replicates per group, and single cell RNA-seq experiments included 3 biological replicates per group.

• Detailed information about number of replicates used per group can be found in the Materials and Methods, figure legends and supplementary files.

• Sequencing data has been uploaded to GEO (GSE145436), as mentioned in the manuscript ‘Data and Materials Availability’ section. The reviewer access link is https://www.ncbi.nlm.nih.gov/geo/query/acc.cgi?acc=GSE145436

and the token is cpwreiqofrabnop.

• We did not encounter outliers.

**Statistical reporting**

- Statistical analysis methods should be described and justified
- Raw data should be presented in figures whenever informative to do so (typically when N per group is less than 10)
- For each experiment, you should identify the statistical tests used, exact values of N, definitions of center, methods of multiple test correction, and dispersion and precision measures (e.g., mean, median, SD, SEM, confidence intervals; and, for the major substantive results, a measure of effect size (e.g., Pearson's r, Cohen's d)
- Report exact p-values wherever possible alongside the summary statistics and 95% confidence intervals. These should be reported for all key questions and not only when the p-value is less than 0.05.

Please outline where this information can be found within the submission (e.g., sections or figure legends), or explain why this information doesn’t apply to your submission:

Detailed descriptions of statistical analyses used in this paper can be found in Materials and Methods. In cases where multiple comparisons issues arose, Benjamini-Hochberg (BH) correction was used.

(For large datasets, or papers with a very large number of statistical tests, you may upload a single table file with tests, Ns, etc., with reference to sections in the manuscript.)

**Group allocation**

- Indicate how samples were allocated into experimental groups (in the case of clinical studies, please specify allocation to treatment method); if randomization was used, please also state if restricted randomization was applied
- Indicate if masking was used during group allocation, data collection and/or data analysis

Please outline where this information can be found within the submission (e.g., sections or figure legends), or explain why this information doesn’t apply to your submission:

Male C57BL/6J wild type mice aged 6 weeks were purchased from The Jackson Laboratory and randomly allocated into control and intermittent hypoxia treatment group. No masking was employed. This information can be found in the Materials and Methods.

**Additional data files (“source data”)**

- We encourage you to upload relevant additional data files, such as numerical data that are represented as a graph in a figure, or as a summary table
- Where provided, these should be in the most useful format, and they can be uploaded as “Source data” files linked to a main figure or table
- Include model definition files including the full list of parameters used
- Include code used for data analysis (e.g., R, MatLab)
- Avoid stating that data files are “available upon request”

Please indicate the figures or tables for which source data files have been provided:

• Figure 1B, C; Figure 1-figure supplement 2 – source data 1

• Figure 2; Figure 2-figure supplement 1 – source data 2

• Figure 3-figure supplement 1,2 – source data 3

• Figure 4; Figure 4-figure supplement 1,2 – source data 4

• Figure 5; Figure 5-figure supplement 1,2,3 – source data 5

• Figure 6; Figure 6-figure supplement 1,2,3,4,5 – source data 6

• Other analysis relevant files are in the Supplementary files 1-4, and are referenced in the Materials and methods.

• Additionally, all raw sequencing data has been uploaded to GEO and information

can be found in the ‘Data and Materials Availability*’* section.
